# Regionalization in elderly care: what makes up a healthcare region?

**DOI:** 10.1108/JHOM-08-2020-0333

**Published:** 2021-12-02

**Authors:** Jitse Jonne Schuurmans, Nienke van Pijkeren, Roland Bal, Iris Wallenburg

**Affiliations:** Erasmus School of Health Policy and Management, Erasmus University Rotterdam, Rotterdam, The Netherlands

**Keywords:** Region, Regionalization, Assemblage, Valuation, Elderly care

## Abstract

**Purpose:**

The purpose of this paper is to explore the formation and composition of “regions” as places of care, both empirically and conceptually.

**Design/methodology/approach:**

This paper draws on action-oriented research involving experiments aimed at designing, implementing and evaluating promising solutions to the entwined problems of a burgeoning elderly population and an increasing shortage of medical staff. It draws on ethnographic research conducted in 14 administrative areas in the Netherlands, a total of 273 in-depth interviews and over 1,000 h of observations.

**Findings:**

This research challenges the understanding of a healthcare region as a clearly bounded topological area. It shows that organizations and professionals collaborate in a variety of different networks, some conterminous with the administrative region established by policymakers and others not. These networks are by nature unstable and dynamic. Attempts to form new regional collaborations with neighbouring organizations are complicated by existing healthcare governance and accountability structures that position organizations as competitors.

**Practical implications:**

Policymakers should take the pre-established partnerships of healthcare organizations into account before delineating the area in which regionalization is meant to take place. A better alignment of governance and accountability structures is also needed for regionalization to occur in healthcare.

**Originality/value:**

This paper combines insights from valuation studies with sociogeographical literature and provides a framework for understanding the assembling and disassembling of “regions”.

## Introduction

Speaking on a Sunday morning news and political commentary programme in early March 2019, the Dutch Minister of Health announced the launch of a regional approach for combatting the increasing problem of staff shortages and inadequate quality in elderly care. Regions, the minister said, would stimulate collaboration among local organizations, practitioners and policymakers. Funds were subsequently made available to elderly care providers to support joint initiatives with neighbouring healthcare organizations focussing on the design and implementation of promising solutions to the problem of medical staff shortages. In addition to requiring organizations to collaborate to obtain these funds, officials from the Ministry of Health used active persuasion to encourage elderly care providers, for example in consultations. Besides these “soft” strategies, the Ministry had no other mandate to enforce regionalization in elderly care. The public (and authoritative) appeal for regional collaboration was surprising in the light of over a decade of market-driven policies in healthcare, which the minister now openly declared to be inadequate to the task of resolving contemporary problems in elderly care. The shift in focus from competition to regional collaboration, however, fits in with a broader (re)discovery of “regions” as places for care ( Lorne *et al.* , 2019 ; Ivanova *et al.* , 2016 ; Jones *et al.* , 2019 ; Oldenhof *et al.* , 2015 ; Lorne *et al.* , 2019 ).

Regions have become popular places for healthcare policymaking, although the trend is not a global one, with some countries seeing a growing level of consolidation and concentration of healthcare providers ( Kapp, 2016 ). The renewed emphasis by policymakers on regionalization more or less implies that regions constitute singular and bounded geographical spaces that are well defined, tangible and “useable” for developing a policy strategy, and it assumes that regional actors feel a sense of regional engagement and a willingness to cooperate in regional care delivery. Regional geographical boundaries are in fact often taken for granted in the organization of healthcare and are seldom a topic of research enquiry. The delineation of regions as primordial and clearly bounded geographical areas is open to question, however, both empirically and conceptually. The socio-political literature on regions shows that they are not just “out there” but emerge from multiple (and often contested) geographical constituencies ( Lorne *et al.* , 2019 ; MacLeod and Jones, 2001 ). Rather than being clearly demarcated, regional boundaries may be fluid or overlap, with multiple (functional, administrative, cultural and professional) regions being contained within the same geographical area.

Increasingly, scholars have come to understand (healthcare) regions as “assemblages” comprising a diverse set of institutional actors tied to provisional allegiances ( Ivanova *et al.* , 2016 ). Rather than a given or based on formal agreements, relations between institutional actors within a regional assemblage are fragile and conflict-ridden. As a result, assembling a region is a continuous process that requires “work” ( Lorne *et al.* , 2019 ). We know little about why such formations are rife with conflict and require continuous maintenance. To fill in this recurrent hiatus in thinking about geographical space relationally, scholars must embrace a richer pallet of conceptual tools that will enable them to understand the “forces that restrict, constrain, contain and connect the mobility of relational things” ( Jones, 2010 , p. 249). In this paper, we take on this challenge. A total of two questions have guided our research: (1) What is a healthcare region? and (2) What mechanisms inform institutional actors' decisions to form, maintain or abandon collaborations with neighbouring organizations within an administrative region?

We draw on a large and ongoing action-oriented research programme in the Netherlands (2019–2021) in which healthcare providers, policymakers and researchers jointly develop and implement solutions to the entwined problems of a burgeoning elderly population and an increasing shortage of staff, particularly of elderly care physicians. In the pilot projects undertaken within this programme, healthcare providers operating within a care office region are encouraged to collaborate on devising and implementing “promising solutions”, such as integrated services, practitioners working for several healthcare providers in the region, shared instruments of triage and shared technological tools. In total, 14 of the 32 care office regions – the administrative area linked to the largest healthcare insurer within a constituency – are represented in the project. The project is innovative in encouraging healthcare providers to cooperate rather than to compete with healthcare organizations in their geographical proximity. As researchers, we study, evaluate and, through our research, contribute to the experiments, elucidating how they impact elderly care and traditional ways of organizing care (e.g. use of triage, integration of GP and nursing home care). By participating actively in healthcare providers' efforts to cooperate regionally, our team obtained an in-depth understanding of the dynamics through which practice and policy networks in elderly care are assembled and disassembled.

We begin this paper by providing a conceptual framework for (healthcare) regions and discuss recent work on socio-spatial formations and valuation practices, a concept that we used to understand the mechanisms shaping institutional actors' work within the networks studied. We then describe our research methods and go on to present our results, arguing that healthcare regions, rather than clearly bounded topological entities, can be better understood as an assemblage comprising a variety of dissimilar actors tied to uneasy allegiances and in a constant state of flux. An analysis of the regimes of valuation embedded in the healthcare sector helps to explain the dynamics through which such socio-spatial formations are assembled and disassembled.

## What is a healthcare region? A conceptual framework

The focus on regions as a valuable site for organizing care has seen a recent upsurge in the medical sociological literature (e.g. Lorne *et al.* , 2019 ). Whereas scholars in this discipline have proposed few theories as to what a healthcare region entails conceptually, a vast body of work on “new regionalism” has emerged in social geography discussing the conceptual understanding of a region and of regionalization ( Agnew, 2013 ; Allen and Cochrane, 2007 ; Amin, 2004 ). We first turn to this body of work for conceptual tools that allow us to grasp what a healthcare region comprises and then move on to the concept of valuation regimes to shed light on the different evaluative principles that underpin processes of regionalization.

In the late 90s, the interest in new regionalism was understood as a reaction to a “failing” centralized state in a globalizing world ( Keating, 1998 ), with regions being seen as a more appropriate bureaucratic level to manage societal challenges and accelerate economic prosperity. New regionalism cemented the idea of regions as more or less delineated geographical areas managed by clearly identifiable politico-administrative institutions ( Macleod and Jones, 2007 , p. 1,180). This conceptualization has also prevailed in health and healthcare research, for instance in quantitative investigations into the effects of place, or the specific conditions of a place, on the health of its denizens ( Hazen and Anthamatten, 2012 ). Such conceptualizations of place have increasingly come under attack, as they falsely assume a bounded topological area with bureaucratic institutions operating on different scalar levels ( Cummins *et al.* , 2007 ). Instead, a relational perspective has been suggested to conceptualize regions and regionalization processes ( Allen and Cochrane, 2007 ; Amin, 2004 ), shifting the analytical focus to the (symbolic) relations between diverse institutional actors that constitute a particular region ( MacLeod and Jones, 2001 ).

Recent work conceptualizes regions as assemblages of diverse sets of institutional actors ( Allen and Cochrane, 2007 ; Lorne *et al.* , 2019 ). This line of work emphasizes the fluidity and heterogeneity of the institutional networks that make up regions and argues against the conceptualization of regions as clearly bounded geographical spaces. Assemblages should be understood as loosely knitted, temporal and entwined relational networks among heterogeneous institutional actors, such as local, regional and central state entities, interest groups and private and public organizations ( Allen and Cochrane, 2007 ; Amin, 2004 ). These spatial-temporal networks are often “problem driven forms of coordination through partnerships and policy exchange” ( Gualini, 2006 , p. 889), meant, for example, to further economic development within a geographical area ( Allen and Cochrane, 2007 ) or in the provision of healthcare. A region, then, can be conceived as a web of relations tied to a provisional allegiance, constantly changing and drawing in institutional actors that operate in various domains and in different geographical constellations.

## (Dis)assembling healthcare region: regimes of valuation

Perceiving a region as an assemblage allows us to scrutinize the ongoing practices of assembling (new) actors, resources and policies and disassembling others to arrange healthcare systems and services into new and meaningful but potentially also conflict-ridden practices of regional care ( Lorne *et al.* , 2019 ). Assemblages are laden with conflictual dynamics; hence, various actors must work to keep the formation together. Lorne *et al.* (2019) , for instance, show that in the context of initiatives to enhance regional collaboration among healthcare providers, austerity triggers a centrifugal dynamic in which healthcare managers prioritize the survival of their own organization above regional collaboration ( 2019 , p. 9). As a result, work is required to sooth the tensions and to keep the formation together. How assemblages come into being and which dynamics galvanize the assembling and disassembling of socio-spatial formations often remain unclear. As Jones (2010) points out, this is a recurrent hiatus in thinking about geographical space relationally, and to fill it in, scholars must embrace a richer pallet of conceptual tools that enable them to understand the “forces that restrict, constrain, contain and connect the mobility of relational things” ( 2010 , p. 249).

The work of (re)assembling socio-spatial formations depends, among other things, on the strategies, interests and actions of actors, for instance whether they value collaboration or prioritize competition. Such decisions are underpinned by certain evaluative principles, cultural logics or sets of norms on which actors draw to critique or legitimize existing arrangements and justify their decisions ( Boltanski and Thévenot, 1999, 2006 ). Managers, for example, often base their strategic decision-making within organizational contexts on a market principle that values self-interest and competition ( Kornberger, 2017 ). It should come as no surprise that such actions often hamper collaboration between organizations. However, as numerous scholars in the field of Science and Technology Studies (STS) have pointed out, the act of valuing is not based solely on ideational cultural logics from which human actors draw their strategic actions and justify and critique arrangements ( Kornberger, 2017 ; Rushforth *et al.* , 2019 ; Zuideren-Jerak and van Egmond, 2015 ). What is rendered valuable is, among other things, produced by concrete practices that are embedded in the organizational infrastructure. In the STS literature, these arrangements are often referred to as “valuation devices” ( Callon *et al.* , 2007 ). Rankings and reviews are pertinent examples; they have pervasive performative effects, trigger certain types of strategic actions and produce ideas of what is valuable ( Kornberger, 2017 ; Rushforth *et al.* , 2019 ; Zuideren-Jerak and van Egmond, 2015 ).

These two strands of valuation literature have opposing views on how to study valuation practices. One body of work focuses on the concrete valuation devices and construes organizational culture as a product of these arrangements ( Kornberger, 2017 ; Rushforth *et al.* , 2019 ). The other strand analyses the cultural logics within organizations and sees valuation devices as an outflow of these evaluative principles ( Fourcade, 2011 ). We draw on the concept of “regimes of valuation” to resolve this conundrum ( Fochler *et al.* , 2016 ). The concept refers to systems of cultural evaluative principles and enmeshed organizational infrastructures, such as valuation devices, organizational routines, discourses and governance and accountability structures that value and galvanize particular strands of strategic action within organizations. Within academia, for instance, the (e)valuation of academic excellence is grounded in a whole organizational apparatus that includes such valuation techniques as citation scores, grants and funding, the number and performance of PhD students, tenure tracks and other organizational routines and the subjectivity of an entrepreneurial self ( Fochler *et al.* , 2016 ; Stark, 2011 ; Rushforth *et al.* , 2019 ). It is this amalgamation of instruments, identities and values that makes up the regime of valuation.

Institutional domains may have multiple regimes of valuation that, at times, contradict one another and trigger conflicting practices of assembling and disassembling networks. Scholars have distinguished between fields in which one evaluative principle is dominant (hierarchies) and fields in which multiple principles coexist in a non-hierarchical relation (heterarchies) ( Stark, 2009 ). This distinction helps to conceptualize the relationship between different regimes of valuation and directs the analysis towards understanding how these regimes interact and to what effect. Writing about valuation practices in academia, Fochler *et al.* (2016) argue that although there are multiple regimes of valuation through which scholars value their work and act strategically, one regime of valuation, which is of competition and productivity, is dominant within the field. The normative power of such a regime is in fact determined by the degree to which it is institutionalized within a particular field ( Fochler *et al.* , 2016 , p. 180). The regime of competition and productivity within academia, for instance, is powerful because it is grounded in a whole infrastructure of citation indexes, grants, hiring procedures, tenure tracks and university rankings. In analysing the strategic decisions actors make in valuing particular chains of action, scholars should consider the whole organizational apparatus in which these evaluative principles are rooted. In this paper, we show that the assembling and disassembling of socio-spatial formations is shaped by multiple regimes of valuation, and that calls for closer regional collaboration are not necessarily effective when a dominant regime of valuation emphasizes competition over collaboration.

## Research methods

The research presented in this paper is part of an ongoing action orientated research. Between November 2018 and July 2020, we conducted a total of 273 semi-structured interviews with managers of nursing homes, elderly care physicians, specialist nurses, welfare workers researchers and policymakers. Respondents participated in various projects aimed at initiating “promising solutions” for the ongoing shortage of medical staff. During the interviews, respondents were asked to reflect on their organization's collaborations, on the organization itself and the quality of elderly care it provided, on initiatives to alleviate the shortage of medical staff and on organizational bottlenecks in current elderly care. Most interviews lasted between 45 and 60 min and were conducted in person. All interviews were audio-recorded with the consent of the interviewees and were transcribed verbatim.

Besides these interviews, we drew data from various ethnographic observations. We attended meetings of regional working groups charged with developing and discussing the various pilots. A variety of different actors participated in these working groups, from healthcare managers and consultants to physicians and nurses. The first pilot in our research project, which ran from January to March 2019, involved four nursing homes experimenting with a nurses' triage model. Over the course of this pilot, two of our researchers shadowed nurses and clinical practitioners (physicians and nurse practitioners) and managers who worked with the triage model. They also participated in the regional project group, providing feedback on their research findings and helping to refine the pilot. The second pilot, which ran from September 2019 to January 2020, reallocated the tasks of an elderly care physician and a general practitioner working in a nursing home in small rural community. In total, two researchers shadowed both professionals in the nursing home over the course of pilot. In total, we conducted over 1,000 h of observations for the whole project.

We analysed our data using an abductive approach ( Timmermans and Tavory, 2012 ). Our research produced “surprising empirical findings” (p. 169) that we analysed against the backdrop of different social theories discussed in the theoretical section above. The first was the dissimilarity between the formal, administrative understanding of a region as a clearly geographically bounded entity, espoused in project plans and policy documents and the actual fuzzy, unbounded spatial formations of collaborating organizations on the ground. Additionally, we found that the process of building partnerships between different actors in the field of elderly care was anything but neat and simple. The partnerships were often fragile and rife with conflict, with participating actors having to juggle differing interests and values, some of which collided, or at times opposing the move towards further collaboration with partner organizations. In the analytic process, we coded our data and “revisited” the phenomena under study, weighing and fitting different theoretical explanations to the aforementioned surprising findings ( Timmermans and Tavory, 2012 ). Additionally, we “defamiliarized” our data by comparing the neat and orderly idiom of regions in policy discourse with the messy, complex and fluctuating web of connections between the actors in our study ( Timmermans and Tavory, 2012 ). We then proceeded to debunk and refine our emerging concepts, comparing our explanations with ill-fitting data and deviant cases. After intensive coding, we identified two “core categories” ( Corbin and Strauss, 1990 ): the composition of socio-spatial formations, referring to the geographical spread of the various practice and policy networks in which our interlocutors operated and the underlying mechanisms of assembling and disassembling these practice and policy networks, referring to the forces that contributed to the formation, maintenance and disintegration of these networks.

## Findings

In the remainder of the paper, we explore the composition of “healthcare regions” empirically and analyse the dynamics of assembling and disassembling practice and policy networks. First, we argue that healthcare regions are not clearly bounded topological entities but should rather be conceptualized as multi-layered webs of relations tied to a provisional allegiance. Some of these webs are formalized in institutionalized networks, and others are informal collaborations in which heterogenous actors, such as healthcare managers, medical professionals, policymakers and consultants, cooperate. This web of relations is constitutively dynamic. Some actors might disconnect from the assemblage while others might join the processes that apply even to the more formalized networks. As a result, the region is always in flux and its geographical reach changes over time. Second, multiple regimes of valuation exist within the healthcare field and shape the dynamics in which of socio-spatial formations are assembled and disassembled.

### The region as a multi-layered assemblage

In the Netherlands, healthcare is delivered by private non-profit and for-profit organizations on a heavily state-regulated healthcare market. Long-term elderly care is covered by the Act on Long-term Care ( *Wet Langdurige Zorg* , *Wlz* ) and is the responsibility of the Ministry of Health. The Ministry has devolved its responsibilities to 32 “care offices” that are linked to the largest healthcare insurer in a particular geographical area. The care offices finance long-term care in a particular area by purchasing care from private, long-term care providers (particularly nursing homes). The care offices also provide funds to organizations in their constituency allowing them to devise promising solutions to the entwined problems of a burgeoning elderly population and a shortage of staff.

Our initial assumption was that the various collaborations between the healthcare providers with which we engaged would be situated largely within the geographical boundaries of the administrative regions covered by the care office. However, this was not the case,. In the first round of interviews, we asked our respondents to delineate their region geographically. It turned out that, by and large, our respondents' understanding of “their region” did not correspond to the care office constituency. Many respondents referred to multiple regions that depended largely on the various existing collaborations in which they and their organizations operated. Some of these collaborations were institutionalized in formal networks of professionals and managers of other, often neighbouring, organizations dedicated to specific themes (e.g. “dementia networks”) or specific arrangements (i.e. stroke care). The institutional actors in our study took part in numerous such collaborations. Other collaborations occurred in practice networks, for instance around particular care paths for the elderly. In this multiplicity of networks, the region was a three-dimensional web of relations spreading out across space. In the following, we illustrate this understanding of the region as a multi-layered assemblage by focussing on our research in *de Achterhoek,* an area on the border between the Netherlands and Germany.

*De Achterhoek* was not an administrative care office region. The care office region to which it belonged (led by the largest healthcare insurer in the region, Menzis) covered a much larger geographical area, including numerous other municipalities in the east of the Netherlands, but excluding two municipalities considered part of *de Achterhoek* in “demotic discourse” (as the discourse of local residents opposed to the “official” idiom of policymakers) ( Baumann, 1996 ) and belonging to the administrative area of another regional care office (led by another insurer). A total of six elderly care organizations in *de Achterhoek* collaborated in designing and implementing a pilot aimed at providing sustainable medical care for the elderly. The nature of these organizations differed from the three large care providers that ran ten or more nursing homes scattered throughout the east of the Netherlands, offering such specialized services as geriatric rehabilitation and observation and diagnosis. The latter three organizations had large medical teams consisting of elderly care physicians, nurse practitioners and paramedics working both intramurally and extramurally. The other participating organizations were smaller care providers with a few locations offering clustered living facilities and without their own medical teams; they depend on collaboration with other nursing homes and general practioners (GP’s) to deliver medical care to their residents. Most of these organizations' facilities were located within the geographical area under the jurisdiction of the Menzis care office. One participating organization formed an exception; it had a large nursing home located in a municipality under another jurisdiction and was not considered part of *de Achterhoek* even in demotic discourse. Unsurprisingly, our team and all our interlocutors had difficulty in grasping what the geographical area of *de Achterhoek* actually was. The collaborations that had emerged historically within formal and informal networks did not necessarily match the geographical boundaries of the care office's administrative “region”.

To make this more concrete, the managers of the different elderly care organizations often met as the “management council”, which also included managers from a hospital (the result of a recent merger between two hospitals in the two largest towns in *de Achterhoek* ), a large organization providing mental healthcare in the east of the Netherlands and a municipal general practitioner association. The management council decided on the funding and continuation of projects developed in various other dependent networks. There were eight of these dependent networks, some focussing on palliative care and care for fragile elderly. Each of these dependent, thematic networks had an *East Achterhoek* and a *West Achterhoek* branch, the legacy of the former division of hospitals in the area ( *de Achterhoek* originally had two hospitals, one in the west and one in the east). The thematic networks were organized around these hospitals and included representatives from healthcare organizations and municipalities that operated in the catchment area of one of these two hospitals. The policy networks, formed around policy programmes ( Kickert *et al.* (1997) , and practice networks, formed around communities of practice ( Addicott *et al.* , 2006 ), in which our interlocutors operated were not conterminous with the care offices' administrative “regions”.

### The region as a dynamic assemblage

The constituencies of these healthcare assemblages were by nature unstable. Provisional allegiances could easily dissolve and new relations form. Such instability also had consequences for the spatial reach of these assemblages, as they fluctuated with the changing institutional relations within networks. Returning to the example of *de Achterhoek,* managers of elderly care organizations participated in a policy network called the “Healthiest Region”, the outcome of administrative collaboration between eight municipalities (spanning a geographical area smaller than the region as understood in demotic discourse). Besides the elderly care organizations, the Healthiest Region network consisted of mental care organizations, a nearby hospital, numerous businesses in the healthcare domain and seven municipalities in *de Achterhoek* . Originally, most of the administrative, formal collaborations between the municipalities in *de Achterhoek* had involved eight stakeholders, including the municipality of *Montferland,* recently formed by the merger between the municipalities of *Bergh* and *Didam.* After the merger, the executive councillors of the new municipality initially sought to collaborate and participate in policy networks with their counterparts and policymakers in the seven other municipalities in *de Achterhoek* . This changed, however, when a new administrative collaboration emerged (SAN) between two larger cities located to the west of *de Achterhoek* that had far more resources. The executive councillors and policymakers of *Montferland* increasingly participated in the new regional SAN networks and withdrew from numerous networks centred around *de Achterhoek,* including the Healthiest Region network. Healthcare initiatives devised in this network were therefore implemented in the seven remaining municipalities. The actors participating in these socio-spatial formations fluctuated over time, depending on administrative changes (merging communities), geographical allegiances and resources. As a result, the geographical reach of these assemblages was in flux.

### Regimes of valuation: understanding the assembling and disassembling of socio-spatial formations

The above examples taken from *de Achterhoek* highlight our understanding of healthcare regions as multi-layered and dynamic assemblages that were compositionally fluid. Actors within the analysed networks continuously assembled and disassembled the socio-spatial formations to which they belonged. This “work” did not occur in a vacuum but, as we will show, emerged from the regimes of valuation embedded in the field of elderly care. These regimes consisted of evaluative principles grounded in infrastructures comprising valuation devices, governance and accountability structures and organizational routines. We delineate four such regimes: the efficiency regime; the market regime, the historic regime and the organizational identity regime, which jointly shaped the formation of the healthcare assemblages (see Table 1 ).

### Assembling the region: the efficiency regime of valuation

The managers and professionals interviewed were almost unanimous in seeing closer collaboration and joint initiatives with colleague organizations as a strategy for maintaining high-quality care in the face of a severe and worsening shortage of medical personnel. This reasoning echoed the main tenet of policy discourses on “regionalization”. Joint initiatives and closer collaboration in elderly care allegedly result in a more economical deployment of scarce resources, such as medical personnel. This logic is what we mean by the efficiency principle, and it is tied to a project-based financial infrastructure. In the following, we illustrate this by focussing on our research in the administrative area *Flevoland.*

In administrative area *Flevoland* , four elderly care providers long had difficulty in delivering high-quality medical care to their clients. They consisted of three larger organizations (Sea-Care, Résidence and Lowlands), which had their own medical teams, and one smaller organization (Island-Care) with a strong religious signature, which – at the start of our research – did not have a medical team and procured these services from Sea-Care. All four organizations were experiencing a severe shortage of medical personnel, especially elderly care physicians, and were among the first to act on the policy calls for closer regional collaboration.

In early 2019, the four organizations launched their first pilot, funded by the regional care office, in which the care teams worked with a new triage model meant to reduce the workload of specialist nurses and elderly care physicians and to streamline the deployment of medical staff within the organizations. It was also hoped that the pilot would lead the organizations to coordinate a variety of work practices, easing staff exchanges and deployment between them and alleviating temporary staff shortages in one with staff from another. A further hope was that the initial project would inspire other initiatives aimed at closer collaboration between the elderly care organizations.

These calls for closer collaboration with neighbouring elderly care providers came with a specific project-based financial infrastructure. When the Netherlands' current government entered office, it reserved 50 million euros for projects targeting specific “regional” bottlenecks in the provision of elderly care. These funds were distributed by the care offices, which required elderly care organizations within their constituency to collaborate in joint projects. As a result, healthcare managers of elderly care organizations began to meet regularly from 2018 onwards to discuss possible joint initiatives addressing the shortage of medical staff. However, these projects had funding for only two or three years, and it was uncertain whether they would continue once the funding dried up. Unsurprisingly, managers of the four organizations in *Flevoland* questioned whether joint initiatives were sustainable in the long run, especially since countervailing forces pushed to disassemble the emerging collaboration.

### Disassembling the region: the market regime of valuation

Over the course of 2019, relations in the elderly care assemblage in *Flevoland* became increasingly strained. The medical staff shortage was intensified, and instead of investing all their efforts in closer collaboration with neighbouring organizations, numerous participants made strategic decisions that were primarily in the interest of their own organization, even at the risk of jeopardizing the provision of care in partner organizations. These decisions were informed by a market principle of valuation emphasizing competition and organizational self-interest ( Boltanski and Thévenot, 2016 ). Again, this principle was not solely ideational; it was enmeshed with the industry's governance and accountability structures and with particular valuation devices that produced the values “self-interest” and “competitiveness”.

The smaller organization Island-Care procured medical care from the larger Sea-Care organization but had long been unhappy with this arrangement. Island-Care's general manager was dissatisfied with the quality of care provided by Sea-Care's medical team. This dissatisfaction was exacerbated by the numerous changes in that team and a current arrangement making a physician assistant (PA) responsible for medical care, albeit under an elderly care physician's supervision. Island-Care's manager claimed that this arrangement jeopardized the quality of care in his facility. The manager of the larger organization, however, argued that PAs were authorized by law to provide medical care within Island-Care's facilities, and that a shortage of elderly care physicians necessitated this arrangement. Since the larger organization was unable to provide an elderly care physician, the manager of the smaller organization considered other strategic options, all based on the premise that an elderly care physician should be responsible for medical care. One of these was a pan-organizational medical team that would be responsible for medical care in all of the nursing homes of the assemblage in *Flevoland.* Ultimately, however, he opted for a different route.

Considering our size, we would like a regional medical team with practitioners and doctors that has our preference, but we have not sat still. Tomorrow, we have a meeting with a professional (an elderly care specialist) who we might hire. Island-Care's manager

Island-Care's manager decided to hire the elderly care physician and terminate its current contract with the larger organization, Sea-Care, much to the displeasure of the latter's general manager and medical staff. The main sore point was the competition for elderly care physicians in their catchment area. The larger organizations were seeking to recruit elderly care physicians themselves and were not amused that the smaller organization, Island-Care, was fishing in the same pond. In the summer of 2019, the three larger organizations decided to exclude the manager of the smaller organization from the management group on regional initiatives for collaboration around the provision of medical care, arguing that his presence hampered the process of regionalization.

This foregrounding of “competitiveness” and “self-interest” did not occur in a vacuum but was underpinned by a governance and accountability structure and by valuation devices through which the aforementioned values were produced. In the Netherlands, nursing homes are required to report annually on the quality of care in their facilities based on certain parameters of “good care”, such as the degree of personalized care, the well-being of clients, patient safety and quality improvement efforts. This form of quality reporting is a “valuation device” and has far-reaching consequences for the type of values embedded in policies and managers' strategic choices ( Zuiderent-Jerak and van Egmond, 2015 ), incentivizing decisions that are primarily concerned with upholding the quality of care within individual facilities, even when they might be detrimental to elderly care in the organization's wider context. Valuation devices are grounded in a particular governance and accountability structure of elderly care. In the Netherlands, the healthcare inspectorate evaluates the quality of care within individual organizations. It actively scouts out organizations that may be providing sub-standard care, and its inspectors may visit and scrutinize individual organizations. If it judges the quality of care in a nursing home to be inadequate, the inspectorate can impose a series of cascading measures intended to improve that quality. This governance and accountability structure takes the individual organization and its locations as the unit of measurement. The structure itself causes managers and medical staff to prioritize the quality of care within the boundaries of their own organization above elderly care in the wider catchment area.

Recent attempts in elderly care to build an infrastructure around the efficiency regime of valuation spurred the assembling of new socio-spatial formations. Another, highly institutionalized regime of valuation, the market regime provoked a countervailing, disassembling dynamic, while yet another regime of valuation, the historic regime, shaped both the assembling and disassembling of networks.

### Assembling and disassembling the region: the historic regime

Elderly care providers often valued the established relations within the assemblage to which they belonged. At times, this complicated the assembling of “the region” as propagated by the Ministry of Health and the care offices. Sea-Care, one of the larger organizations in administrative region *Flevoland* , not only had nursing homes in *Flevoland* but also in another care office region, *Friesland.* In recent years, Sea-Care’s management board had invested heavily in its partnerships with two elderly care organizations, Aquarius and Pisces, both of which had nursing homes in *Flevoland* but whose core operations were located in *Friesland.* The four elderly care organizations in *Flevoland* had been discussing preliminary plans for a pan-organizational medical team that would care for all four's clients, but this potential collaboration compromised the strategic plans of Sea-Care's management board.

Yes, this pan-organizational medical team could be one of the outcomes, but what should we do with all our strategic plans? We are also collaborating with Aquarius and Pisces on rehabilitation and palliative care. Sea-Care's manager

Valuing the established partnerships in this way complicated relations between the institutional actors in *Flevoland* . On the one hand, Sea-Care's general manager was convinced that regionalization and mutual cooperation were needed to address the challenges elderly care organizations were facing. On the other hand, he was questioning whether to seek solutions with the three other parties in *Flevoland* , since he had established partnerships with organizations outside this administrative area. His hesitation was noted by the managers of the three other organizations in *Flevoland* , who questioned Sea-Care's allegiance. The valuing of historic relations complicated the emergence of collaborations in the various constituencies of the care offices, with actors often prioritizing relations with organizations from another administrative region.

The valuing of historic relations also benefitted the assembling of healthcare regions, although this seldom involved all the elderly care providers in a care office region. The *Friesland* care office region was one example *.* In late 2018, one larger elderly care provider, About-Care, suggested setting up a pan-organizational medical team composed of elderly care physicians, specialist nurses and paramedics. The other *Friesland* organizations did not support the plan, however, and there was no history of collaboration to fall back on. About-Care then sought closer collaboration with their established partners, including a nearby hospital and a mental health provider. These parties drew up a new plan to create a pan-organizational medical team of elderly care physicians, psychiatrists and geriatricians who would provide care in all three organizations. The plan had the support of the care office. Valuing historic relations could thus both spur as well as hamper the formation of care assemblages.

### Assembling and disassembling the region: the organizational identity regime

Yet another regime of valuation shaped the configuration of the assemblages in elderly care. Some actors valued what they saw as the idiosyncratic signature of care in their organization, and this had consequences for the partnerships they formed. We saw this when studying a stand-alone nursing home in the middle of the Netherlands. Like most stand-alone nursing homes, it could not afford its own medical team and procured medical services from a for-profit elderly care provider. The elderly care physicians working in the stand-alone facility rotated, much to the dismay of its care workers and management, who took pride in being a small-scale organization offering lifelong employment in which care providers were accessible and in which clients and staff knew one another personally. Valuing these traits meant devaluating the partnership with the for-profit organization. Using the funds available for regional collaboration, the management of the stand-alone facility explored opportunities for a different arrangement of medical care, one that would support their own values of care and allow them to maintain their independence. The solution was to collaborate more closely with a local primary care organization, whose affiliated general practitioners would provide basic medical care in the nursing home with the support of an elderly care physician made available by the for-profit organization. The stand-alone facility and the local GPs valued this new collaboration, since it meant continuity of care for their clients, a value that both parties saw as fundamental to the care they provided.

In the previous example, the valuing of the organization's idiosyncratic signature of care resulted in both the disassembling and assembling of care arrangements. This regime of valuation existed alongside the efficiency, market and historic regimes, with the action spurred by and the interaction between these regimes shaping the assembling and disassembling of socio-spatial formations in elderly care.

## Conclusion

Our paper challenges the prevailing understanding of (healthcare) regions as clearly bounded topological areas, a view common in policy circles and healthcare research ( Hazen and Anthamatten, 2012 ). We asked, what is a healthcare region? Drawing on socio-geographical insights, we regard a healthcare region as a complex, dynamic and multi-layered assemblage of heterogeneous – public and private, administrative and professional, national, regional and local – institutional actors ( Allen and Cochrane, 2007 ; Amin, 2004 )). We build on recent studies focussing on the spatial dimension of healthcare restructuring ( Lorne *et al.* , 2019 ) and emphasize the multi-layeredness, dynamism and unbounded nature of healthcare regions. The actors in our study participated in a variety of policy and practice networks that were entangled in complex ways and together formed the “regional assemblage” in which regional care initiatives were established. This assemblage was dynamic, with the composition of the constituent networks – even the mandated ones – changing over time and therefore challenging the general understanding of such networks as being rather rigid, with prescribed roles and fixed membership ( Waring *et al.* , 2017 ). Spatially, this healthcare assemblage was boundless and unstable. It comprised a multitude of networks with a diverse topological reach that changed as institutional actors joined or left. As a consequence, there is no unequivocal reading of what the healthcare region is; instead, it must be conceptualized as an entangled web of networks that is compositionally dynamic.

Regionalization fits in with a larger trend in which governance through networks is seen as a potentially productive approach for addressing “wicked problems” ( Ferlie *et al.* , 2013 ). Recent scholarship emphasizes the discrepancy between the managerial and technocratic view of networks as entities that can be mandated and imposed and a more sociological understanding of networks as emerging from the sustained and meaningful interaction of actors ( Waring *et al.* , 2017 ). We recognize this tension in our research. The policymakers and care offices we studied had a rather technocratic understanding of networks as entities that could be mandated and imposed on the field. As authorities, they set the parameters for regionalization as interorganizational collaborations meant to emerge within a given constituency. At times, this conflicted with the relevant organizations' established networks; some (like Sea-Care) had established partnerships with organizations in a different constituency, and this affected their commitment to the new, mandated networks. At the same time, these new, mandated networks jeopardized established partnerships when managers started to question which strategic partnerships would be viable in the long run. This echoes other research showing that the establishment of mandated networks is often complicated by pre-established interorganizational relations ( Waring *et al.* , 2017 ), and that such networks often threaten pre-established practice networks ( Addicott *et al.* , 2006 ). In this light, policymakers would benefit from having a more fluid understanding of what constitutes a region and from acquiring a more sociological perspective on networks. They might wish to refrain from *a priori* delineation of the geographical area and organizations but instead leave regionalization to the field and allow it to emerge from established relations within a healthcare assemblage.

It has been suggested that governance in networks can overcome the fragmentation brought about by the marketization of healthcare ( Ferlie *et al.* , 2013 ). Networks would foster relations of trust, openness, cooperation, shared decision-making and resource sharing, even if underlying market structures exist ( Kickert *et al.* , 1997 ). Other scholars, however, argue that network relations are often far from harmonious. They are shaped by structural power dissymmetries between groups and relations can be conflictual, for instance due to inconsistencies in health policies that push and pull actors within the network in opposite ways ( Waring *et al.* , 2017 ; Addicott *et al.* , 2006 ). We asked the following: what mechanisms inform institutional actors' decisions to form, maintain or abandon collaborations with neighbouring organizations within an administrative region? Our paper offers a framework for understanding the conflictual dynamics within policy networks. Rather than seeing strained relations within networks as the product of different sets of values ( Klijn and Teisman, 2003 ), we argue that tensions may arise from conflictual valuation regimes within the healthcare sector. One practical implication of our analysis is that calls for regionalization in healthcare will be ineffective unless the underlying governance and accountability structures are addressed. Current governance and accountability structures of elderly care in the Netherlands are tied to a market evaluative principle, meaning that managers value strategies that are primarily in the interest of their own organization. Without changing these underlying structures, the objectives of regionalization are unlikely to be realized. New modes of accountability that work from a networked perspective must be developed to make regionalization work.

## Figures and Tables

**Table 1:** Summary of regimes of valuation and the consequences for regionalization

Evaluative principle	Evaluative logic (what is valued?)	Structurally embedded in	Consequences for regionalization
Efficiency	An economical deployment of scarce resources	Project-based financial infrastructure	Assembles regions
Market	Self-interest and competition	Valuation devices, governance and accountability structures	Disassembles regions
Historic	Established partnerships	Policy and practice networks	Assembles and disassembles regions
Organizational identity	Idiosyncratic signature of the organization	Organizational routines	Assembles and disassembles regions
